# Comparative Analysis of Open-Source Tools for Conducting Static Code Analysis

**DOI:** 10.3390/s23187978

**Published:** 2023-09-19

**Authors:** Kajetan Kuszczyński, Michał Walkowski

**Affiliations:** Department of Telecommunications and Teleinformatics, Wroclaw University of Science and Technology, 50-370 Wroclaw, Poland

**Keywords:** SAST, security, vulnerabilities, code analysis

## Abstract

The increasing complexity of web applications and systems, driven by ongoing digitalization, has made software security testing a necessary and critical activity in the software development lifecycle. This article compares the performance of open-source tools for conducting static code analysis for security purposes. Eleven different tools were evaluated in this study, scanning 16 vulnerable web applications. The selected vulnerable web applications were chosen for having the best possible documentation regarding their security vulnerabilities for obtaining reliable results. In reality, the static code analysis tools used in this paper can also be applied to other types of applications, such as embedded systems. Based on the results obtained and the conducted analysis, recommendations for the use of these types of solutions were proposed, to achieve the best possible results. The analysis of the tested tools revealed that there is no perfect tool. For example, Semgrep performed better considering applications developed using JavaScript technology but had worse results regarding applications developed using PHP technology.

## 1. Introduction

The digital transformation continues to accelerate. More and more businesses and regular users are utilizing various types of software, whether for work or entertainment. Access to public services is also undergoing digitization processes. For instance, in Poland, the development of the mObywatel application enables the storage of identification documents in mobile applications [1], electronic circulation of medical prescriptions (E-recepta) [2], and electronic tax filing (e-PIT) [3]. Newer and larger systems are being developed, comprising thousands of lines of code, and numerous libraries and technologies. A steadily growing number of programmers collaborate on a single project, needing to work closely together to deliver a finished product on time. An increasing number of services are available over the Internet, and they also have extended functionality. In summary, the impact of software security also affects the end user, regardless of the device they use, such as the Internet of things (IoT), sensor-equipped devices, embedded systems, or a mobile phone [4]. The importance of securing such software applications, which frequently involve complex codebases, cannot be overstated. Vulnerabilities in these applications can lead to serious security breaches, data leaks, and even physical harm, if the devices controlled by the software are critical to safety.

Cybercriminals tirelessly devise new ways to exploit vulnerabilities in application functioning, to cause harm and extract data. Analyzing program code for security purposes is challenging, time-consuming, and expensive. Thus, there is a necessity to support these processes. Examples of such solutions include tools for conducting static code analysis for security (SAST), and dynamic code analysis for security (DAST). Both of these processes have been discussed in the literature [5,6]. However, it can be observed that authors tend to focus on only one technology, such as C [7,8] or Java [6]. The literature also includes comparisons of solutions for related technologies, such as C and C++ [9]. Additionally, the authors in [6] used enterprise-type tools that are not available to every user due to their high cost. According to the current state of the authors’ knowledge, there is a lack of a broader comparison of open-source tools available to everyone, supporting current trends in software development. Furthermore, the literature lacks a perspective on solutions that can perform static code analysis for more than one technology, such as analyzing code written in both Java and JavaScript. Such solutions can potentially reduce the number of tools used in the software development process, thereby simplifying the continuous integration/continuous delivery process and reducing the amount of data processed in the big data process [10].

The novel contribution of this paper relates to the comparison of the results of open-source tools supporting various technologies used in software development for conducting static analysis and detecting potential errors affecting the security of applications, thereby enhancing the security of organizations and end users. Based on the analysis of the obtained results, a recommendation was formulated regarding the utilization of such solutions, which could significantly enhance the quality of applications developed, even at the code-writing stage. The analysis was carried out based on the list of vulnerabilities reported by all tools. Vulnerable web applications were scanned using these tools for the most popular programming languages [11].

The scope of this work encompassed a review of the available literature; an analysis of methods for scanning code for vulnerabilities; a determination of the pros and cons of SAST tools; an overview of existing tools, their comparison, research, and analysis of the obtained results; as well as effectiveness verification. Within the conducted research, vulnerable web applications were configured and launched, SAST tools were configured and launched, the extraction, transformation loading (ETL) process [12] was utilized to consolidate results from the tools, and the acquired data were processed. Given that web applications are the most popular type of application enabling access to services through personal computers, this work specifically focused on them. This paper is divided into the following subsections:

- Background—describes the research basics and presents the problems, processes, and compromises that occur in static code analysis research;
- Environment—describes the hardware and software used for conducting the research. This section also provides an overview of the analyzed tools and the utilized vulnerable web applications;
- Research Methodology—covers the design of the experiment for the research conducted;
- Results—provides a discussion of the results, and highlights the strengths and weaknesses of the examined tools for static code analysis;
- Conclusions—summarizes the obtained results.

## 2. Background

A web service is a component of an information technology system that can be communicated with via the Internet. This could be a web application, an API server, and so on. With the ever-improving global access to fast and affordable internet connections, these services have become more popular than ever before—they are used for tasks such as online banking, information searches, doctor appointments, watching movies, listening to music, and playing computer games. However, not only has their popularity increased, but their functionality has also expanded. Bank websites offer more and more features, government websites enable administrative procedures—all through the use of computers or smartphones.

Nevertheless, despite the many advantages of web services, as their complexity grows, so does the likelihood of vulnerabilities that could lead to security threats. In the context of cybersecurity, a vulnerability (or flaw or weakness) is a defect in an information technology system that weakens its overall security. Exploiting such a flaw, a cybercriminal could cause harm to the system owner (or its users) or pave the way for further attacks on the system. Each vulnerability can also be described as an attack vector—a method or means that enables the exploitation of a system flaw, thereby jeopardizing security. A set of attack vectors is termed the attack surface [13]. The utilization of a vulnerability is referred to as exploitation.

The common weakness enumeration (CWE) is a community-developed list of software and hardware weakness types [14]. As of today, the CWE database contains 933 entries [15]. Each CWE entry possesses attributes that describe the specific weakness, including an identification number, name, description, relationships to other entries, consequences, and examples of exploitation.

The common vulnerability scoring system (CVSS) is a standard for rating the severity of vulnerabilities [16]. It aims to assign a threat rating to each identified vulnerability (in the case of a successful exploit). Such a rating allows prioritizing the response efforts of the reacting team based on the threat severity. The latest version is CVSSv3.1, released in June 2019. The specification is available on the website, and CVSSv4 is currently in the public consultation phase [17].

The common vulnerabilities and exposures (CVE) database contains entries about known security vulnerabilities [18]. It differentiates vulnerabilities that can directly lead to exploitation from exposures that may indirectly lead to exploitation. Each entry is assigned a unique identifier and includes a description, name, software version, manufacturer, cross-references to other resources about the entry, and creation date.

Due to the increasing complexity of web systems, software testing for security vulnerabilities has become an essential and critical activity in the software development life cycle (SDLC), especially for web applications [19]. Secure software development life cycle (SSDLC) is an extension of the SDLC, with additional security measures [20]. It aims to assist developers in creating software in a way that reduces future security threats. It includes, among other things, defining and implementing security requirements alongside the functional requirements of the application being developed, as well as periodically assessing the security level, for instance, through conducting penetration tests.

Numerous SSDLC models have been proposed and are successfully employed in contemporary processes [20]. Some of these include the National Institute of Standards and Technology (NIST) guidelines 800-64 [21], Microsoft’s Security Development Lifecycle (MSSDL) [22], and the Comprehensive Lightweight Application Security Process by OWASP (OWASP CLASP) [23].

Penetration testing is one of the most popular methods for assessing the security level of web applications [24]. It constitutes a part of the testing phase within the SSDLC process. It involves attacking the application to discover the existence or extent of vulnerabilities within the application’s attack surface. In contemporary cybersecurity solutions, automation plays a pivotal role. As the complexity of developed solutions continues to grow, the need for more efficient methods of testing the security of web services arises. In today’s fast-paced environment, where software updates are released daily, penetration tests must be conducted swiftly and effectively.

It is impossible to fully automate the entire process of conducting penetration tests—certain aspects must be carried out by a human. However, many tasks, such as fuzzing (a technique involving the supply of incorrect, unexpected, or random data), can be easily automated. Although no automation tool can fully replace the intuition and abstract thinking of a human tester, it can expedite their work by identifying well-known and documented vulnerabilities.

Static code analysis tools analyze the source code of a program, utilizing a white-box testing approach. There are various approaches to conducting this analysis, such as string pattern matching, lexical analysis, and abstract syntax tree (AST) analysis [25]. The earlier a software error is detected, the lower the cost of its resolution [26]. SAST tools can scan code not only during the software testing phase within the SDLC but also during the writing of the program code by the developer, providing real-time error feedback. They examine the entire code, ensuring 100% coverage of the software. Besides detecting vulnerabilities in the code, these scanners often analyze the libraries used by the application, highlighting those with known vulnerabilities. Despite developers’ positive evaluation of using SAST tools for error reduction, integrating such tools into SDLC processes encounters certain challenges, such as low analysis performance, the need for multiple tools, and technical debt (a phenomenon where choosing a seemingly easier and cheaper option in the short term becomes less cost-effective in the long run [27]) when implemented late [28]. Scanning extensive lines of code can result in hundreds or even thousands of vulnerability alerts for a single application. This generates numerous false positives, prolongs investigation time, and diminishes trust in SAST tool results [28].

Unlike SAST tools, dynamic application security testing (DAST) tools assess the behavior of running program code through the user interface and APIs. This is a black-box approach to penetration testing, as they lack access to the source code of the tested application. DAST tools are language-independent and can identify issues not present in the application code, such as misconfigured environments, manipulation of cookies (text snippets stored by a web browser in a computer’s memory—can be transmitted by a web service or created locally), and errors in integration with third-party services [5]. As DAST tools do not have access to the source code of the tested application, there is a significant likelihood that they may overlook certain parts of the scanned application. They will not pinpoint the location of vulnerabilities in the code; rather, they will indicate the detected issues, leaving it up to the programmer to identify the line(s) of code responsible for the error.

Since DAST requires a functioning application, vulnerabilities are detected towards the end of the SDLC process, increasing the cost of their remediation. Additionally, a separate environment is needed for conducting tests, further amplifying the financial investment—the entire infrastructure of the tested service must be provided, typically encompassing (but not limited to) the client application, API server, and database. Similarly to SAST tools, they also generate numerous false alarms.

Static and dynamic code analysis for security are not the only types of code analysis. Additionally, we cab distinguish the following: interactive application security testing (IAST), a method that combines SAST and DAST. It utilizes monitoring mechanisms to observe the behavior of the web application server’s code, while simultaneously attacking the application through its graphical interface or API [5]; runtime application self-protection (RASP), a solution that involves using tools to monitor the behavior of a web application during runtime to detect and block attacks. Unlike IAST, RASP does not attempt to identify vulnerabilities but rather protects against attacks that might exploit those vulnerabilities.

## 3. Environment

To ensure accurate research results, an appropriate testing environment was prepared. It consisted of computer hardware and software that enable a thorough analysis process. In order to facilitate seamless reproduction of the experiment results, open-source software and licenses allowing their use for research purposes were utilized. Table 1 presents a list of the hardware and software used to conduct the research.

### 3.1. Tested Vulnerable Web Applications

Table 2 presents the tested web applications. These applications were designed to include numerous security vulnerabilities. Many of them also come with a list of present vulnerabilities, which facilitates the analysis of the results from static code analysis tools.

- EasyBuggy—Prepared by Kohei Tamura, EasyBuggy is a deliberately flawed web application, written to understand error behavior and vulnerabilities [29], using the Java programming language. A list of implemented vulnerabilities is available on the project’s website;
- Java Vulnerable Lab—Created by the Cyber Security and Privacy Foundation (CSPF), the Java Vulnerable Lab is intended for Java programmers and others interested in learning about vulnerabilities in web applications and how to write secure code [30];
- SasanLabs VulnerableApp—Another application written in Java, VulnerableApp is a project prepared by and for cybersecurity experts [31]. According to the creators, it stands out for its ability to be extended by individuals who want to utilize it. The project is part of the OWASP Foundation’s incubator;
- Security Shepherd—This application was prepared by the OWASP foundation, in Java language. It serves as a platform for training skills in the field of cybersecurity. It was designed to enhance awareness of application security [32];
- Broken Crystals—Developed by Bright Security, using JavaScript (and TypeScript), Broken Crystals is an application that implements a collection of common security vulnerabilities. The list of vulnerabilities can be found on the project’s website [33];
- Damn Vulnerable Web Services—An application created by Sam Sanoop using JavaScript. It facilitates learning about vulnerabilities related to Web Services and APIs [34]. The list of present vulnerabilities can be found on the project’s website;
- OWASP Juice Shop—Described as possibly the most modern and sophisticated insecure web application [35], it was developed by the OWASP Foundation for practicing cybersecurity skills. The list of vulnerabilities can be found on the project’s website [36];
- OWASP NodeGoat—Developed by the OWASP Foundation, NodeGoat is an application designed to provide an environment for learning how the vulnerabilities described in the OWASP Top 10 relate to web applications developed using Node.js technology [37];
- Conviso Vulnerable Web Application—Developed by Conviso, this vulnerable web application, written in PHP, simulates an internet blog created by an inexperienced programmer who has made many mistakes, making it vulnerable to various attacks [38];
- Damn Vulnerable Web Application—This web application, written in PHP, is intended to assist cybersecurity professionals in testing their skills and tools in a controlled environment. It can also help web application developers better understand the process of securing their applications [39];
- WackoPicko—Created for a scientific article [40], this web application written in PHP is designed to test vulnerability scanners for web applications. A list of vulnerabilities can be found on the project’s website [41];
- Xtreme Vulnerable Web Application—This improperly written PHP web application helps cybersecurity enthusiasts learn about application security [42]. The list of vulnerabilities can be found on the project’s website;
- Damn Small Vulnerable Web—A small, vulnerable web application written for educational purposes in less than a hundred lines of Python code. It includes most of the popular vulnerabilities, along with examples of exploitation [43];
- Damn Vulnerable GraphQL Application—This vulnerable web application, written in Python, improperly implements GraphQL technology, to teach about its security. A list of vulnerabilities can be found on the project’s website [44];
- Damn Vulnerable Python Web Application—Inspired by the Damn Vulnerable Web Application, this vulnerable web application written in Python aims to create a realistic, vulnerable web application with as many vulnerabilities as possible [45];
- Tiredful API—This vulnerable web application, created using Python, aims to educate programmers, testers, and cybersecurity specialists about web application vulnerabilities [46].

### 3.2. Selected Code Analysis Tools

Within the scope of this study, 11 code analysis tools were tested. For SAST tools, both those dedicated to specific technologies (e.g., Java) and those supporting multiple technologies were chosen. Table 3 presents the tools used for code analysis. Table 4 presents the support of the selected SAST tools for various technologies. All chosen tools operate locally on a computer. They are not cloud-based solutions, although some of them can be incorporated into the continuous integration/continuous delivery (CI/CD) process—a software delivery method that introduces automation into application deployment stages. The employed tools differed in aspects such as their supported technologies, extensibility capabilities, and the content of generated reports.

- Bearer CLI—This is a SAST tool. Currently, it supports JavaScript, TypeScript, and Ruby languages. It does not support adding new rules (one would need to download the source code, add rules, and compile the program), but it allows disabling existing ones and excluding a specific file (entirely or partially) from the scanning process. The report can be generated in three formats: JSON, YAML, and SARIF. Each entry in the report includes the file path, line number of the code, and a description of the detected vulnerability (along with the CWE identifier(s)) [47];
- phpcs-security-audit—Prepared by Floe, phpcs-security-audit is a set of rules for the PHP_CodeSniffer tool. It extends its capabilities for detecting vulnerabilities and weaknesses in PHP code, turning it into a SAST tool. The scan report is displayed in the system shell console window. It includes file paths, line numbers of the code, and descriptions of detected vulnerabilities, though without any links to, for example, the CWE database [48];
- Graudit—Developed by a single programmer, Eldar Marcussen, Graudit is a SAST tool that searches for potential vulnerabilities in application source code, using another tool, GNU grep, for text filtering. It supports multiple programming languages (including all those tested within this work—a complete list can be found on the project’s website). It is essentially a Bash script. Adding rules involves entering specific rules into files provided with the tool. Similarly, you can “disable” certain rules by removing them. The tool’s output is text displayed in the system shell console. It contains only file paths, the line numbers of the code, and highlighted code snippets that triggered the rule [49];
- Insider CLI—This is a SAST tool prepared by InsiderSec. It supports Java, Kotlin, Swift, C#, and JavaScript languages. It does not allow extending the set of rules, but specific files or folders can be excluded from scanning. The report, available in HTML or JSON format, includes a list of detected vulnerabilities. Each vulnerability is accompanied by a CVSS score, file path, line number of the code, description, and removal recommendation [50];
- Find Security Bugs—This is a plugin for the SpotBugs tool, supporting web applications written in the Java language. Similarly to phpcs-security-audit, it extends its capabilities to identifying security vulnerabilities, effectively turning it into a SAST tool. The report is generated in HTML or XML format. Each entry in the report includes the file path, identifier of the detected vulnerability (which, when entered on the tool’s website, provides a detailed description of the vulnerability), and line number of the code [51];
- Progpilot—This is a SAST tool for scanning code written in the PHP language for security vulnerabilities. It allows adding custom rules, disabling existing ones, and excluding files and folders from scanning. The JSON format report contains a list of vulnerabilities, each having various attributes, such as the file path, line number of the code, type, and a link to CWE [52];
- Bandit—This SAST tool, developed by PyCQA, is designed for scanning code written in the Python language for security vulnerabilities. It allows adding custom rules, disabling existing ones, and excluding files and folders from scanning. The report can be generated in various formats such as JSON, CSV, or HTML. For each detected vulnerability, details are included, such as the file path, line number of the code, vulnerability type and description, along with the CWE identifier [53];
- Semgrep—Semgrep, a versatile SAST tool supporting multiple programming languages, allows you to add custom rules, disable existing ones, and exclude specific files and folders from scanning. Reports can be generated in various formats, such as JSON or SARIF. For each vulnerability, details are provided, including the file path, line number of the code, vulnerability type and description, along with references to resources such as the CWE and OWASP Top 10 [54];
- Scan—The SAST tool developed by ShiftLeft supports multiple programming languages (full list available on the project’s website). It is not a standalone solution; Scan is a combined set of static analysis tools. The scan report is a combination of reports from the tools used by Scan. It does not allow for rule disabling or excluding specific files or folders. The report is distributed across multiple files in formats such as HTML, JSON, and SARIF. Each entry in the report contains information about the vulnerability’s location and type [55];
- Aura—The SAST tool developed by SourceCode. AI is used for scanning code written in the Python programming language. The tool does not allow adding custom rules, nor can individual rules be blocked. The report can be generated in text, JSON, or SQLite database file formats. Each detected vulnerability is associated with a file path, line number, and vulnerability type [56];
- Horusec—A SAST tool for scanning code written in multiple programming languages. It uses its own scanners, as well as other open-source ones (a full list is available in the documentation). It allows adding custom rules and excluding specific files and folders from the scan. The report can be saved in text, JSON, or SonarQube formats. Each entry in the report contains a description of the detected vulnerability (not always with a CWE identifier) and its location [57].

## 4. Research Methodology

Table 5 presents a structured summary of experiments conducted in this paper. Each row represents a distinct software application and each column corresponds to a specific SAST tool. The letter “Y” indicates that the experiment was conducted using the given tool and application. A total of 95 experiments were conducted as part of the research.

### 4.1. Data Unification

Each tool presented results in a unique way. To conduct further analysis, it was necessary to unify the received data. For this purpose, Python scripts were written to implement the ETL process: they extracted data from reports written in different formats, modified them to a predefined format, and then output the reformatted data to CSV (comma separated values) files. A separate CSV file was created for each application. The file contained data in tabular form, where each row represented a vulnerability detected by a specific tool. The columns in the file included

- the name of the tool that detected the vulnerability;
- the vulnerability type assigned by the tool;
- vulnerability location—file name;
- beginning of vulnerability in code—line number;
- end of vulnerability in code—line number;
- description of vulnerability provided by the tool;
- evidence of vulnerability provided by the tool;
- additional information provided by the tool.

Not all tools provided complete information. For example, Graudit did not specify the vulnerability type. In such cases, the corresponding fields for vulnerabilities remained empty.

### 4.2. Used Indicators

To perform appropriate measurements regarding the effectiveness of vulnerability detection in the analyzed applications using selected SAST tools, a methodology and metrics proposed by the OWASP organization were utilized [58]. However, their recommended tool was abandoned because it focuses solely on one technology and does not allow checking various vulnerability variants that may occur in the application code. Therefore, for each tool, a set of indicators was calculated:

- True Positive (TP)—Indicates the count of vulnerabilities reported by the tool that actually exist in the corresponding location within the application’s code (also referred to as correctly reported vulnerabilities);
- False Positive (FP)—Represents the count of vulnerabilities reported by the tool that do not actually exist in the corresponding location within the application’s code (also referred to as incorrectly reported vulnerabilities);
- True Negative (TN)—Represents the count of vulnerabilities that were not reported by the tool and do not exist in the corresponding location within the application’s code (also referred to as correctly not reported vulnerabilities);
- False Negative (FN)—Represents the count of vulnerabilities that were not reported by the tool but actually exist in the corresponding location within the application’s code (also referred to as incorrectly not reported vulnerabilities);
- Positive (P)—Indicates the number of vulnerabilities reported by all tools that exist in the application. The P indicator is calculated using Equation (1). This is the same for all tools within a given application.
  (1) P=TP+FN
- Negative (N)—Represents the number of vulnerabilities reported by all tools that do not exist in the application. The N indicator is calculated using Equation (2). It is the same for all tools within a given application.
  (2) N=FP+TN
- TOTAL—This indicator represents the total number of vulnerabilities reported by all tools. It is the same for all tools within a given application.
- Accuracy (ACC)—Accuracy evaluates the portion of all vulnerabilities reported by all tools that consist of correctly reported vulnerabilities, as well as correctly unreported vulnerabilities using a specific tool. The ACC indicator is determined using Formula (3).
  (3) ACC=(TP+TN)/TOTAL
- Sensitivity (SEN)—Sensitivity assesses the proportion of correctly reported vulnerabilities among all reported vulnerabilities. The SEN indicator is calculated using Formula (4).
  (4) SEN=TP/P=TP/(TP+FN)
- Precision (PRE)—Precision evaluates the proportion of correctly reported vulnerabilities among all reported vulnerabilities. The PRE indicator is calculated using Formula (5).
  (5) PRE=TP/(TP+FP)
- TP%—The TP% indicator determines what proportion of vulnerabilities reported by all tools are correctly reported vulnerabilities;
- FP%—The FP% indicator determines what proportion of vulnerabilities reported by all tools are incorrectly reported vulnerabilities;
- TN%—The TN% indicator determines what proportion of vulnerabilities reported by all tools are correctly not reported vulnerabilities;
- FN%—The FN% indicator determines what proportion of vulnerabilities reported by all tools are incorrectly not reported vulnerabilities.

## 5. Results

The study conducted in this work examined 11 SAST tools. These tools were used to scan 16 vulnerable web applications written in four different technologies. The tools and applications are presented in Section 3.

### 5.1. Results for Applications Developed Using Java Technology

Table 6 presents a comprehensive analysis of the various static application security testing (SAST) tools for the EasyBuggy application. These tools were evaluated based on the indicators presented in Section 4. Among the tools examined, Semgrep performed the best in terms of true positives (TP), with eight identified vulnerabilities. This indicates that Semgrep was effective in detecting actual security issues within the EasyBuggy application. However, it is worth noting that FindSecBugs achieved the highest sensitivity (SEN) at 87.88%. This means it had a higher capability to identify true positives relative to the other tools, even though the absolute number of TP was lower compared to Semgrep. On the other hand, Graudit had no true positives (TP) in this context, which raises concerns about its effectiveness for this specific application. It is important to consider that the absence of TP could indicate either a lack of vulnerabilities in the code or limitations in the tool’s scanning capabilities. In terms of false positives (FP), Horusec had the highest count, with 38. High FP values can lead to wasted resources and time investigating false alarms.

Table 7 provides a comprehensive analysis of the various SAST tools applied in the Java Vulnerable Lab application. The results reveal important insights into each tool’s performance:

- TP—Among the tools, FindSecBugs achieved the highest count of TP, with 32 vulnerabilities detected. This indicates a strong capability to identify actual security issues within the application;
- FN—Each tool had varying counts of false FN, representing missed vulnerabilities;
- FP—Horusec had the highest count of False FP with 38, which could lead to resource-intensive investigations of false alarms;
- TN—The tools also correctly identified true TN, where no vulnerabilities were present;
- ACC%—The overall accuracy of the tools ranged from 22.02% (Graudit) to 94.50% (FindSecBugs), indicating their effectiveness in correctly classifying vulnerabilities;
- SEN%—FindSecBugs achieved the highest SEN at 96.97%, indicating its strong capability to identify true positives relative to the other tools;
- PRE%—ShiftLeftScan had the highest PRE, at 87.50%, suggesting that when it reported a positive result, it was often a true positive;
- TP%—The proportion of actual vulnerabilities detected by each tool varied, with FindSecBugs achieving 29.36%;
- FN%—The proportion of missed vulnerabilities ranged from 0.92% (Graudit) to 29.36% (Horusec);
- FP%—The proportion of false alarms varied, with Graudit having a high FP rate of 64.22%;
- TN%—The proportion of correctly identified non-vulnerable instances varied but was generally high for all tools.

Table 8 presents a comprehensive analysis of the various SAST tools applied for the Security Shepherd application. The results provide valuable insights into the performance of each tool:

- FindSecBugs detected the highest number of TP, with 196 vulnerabilities identified. This indicates a strong ability to uncover actual security issues within the application;
- Graudit had a considerably lower number of TPs (19), which suggests it may have missed several vulnerabilities within the application;
- Horusec detected 177 TP, indicating a good capability to identify security issues;
- Insider identified only six TP, signifying limited effectiveness in detecting vulnerabilities;
- ShiftLeftScan found 173 TP, demonstrating a robust ability to identify security problems;
- Semgrep detected 28 TP, indicating some effectiveness in identifying vulnerabilities;
- FP was highest for FindSecBugs with 767, followed by Horusec with 801. These high FP counts could lead to resource-intensive investigations of false alarms;
- The tools correctly identified TN where no vulnerabilities were present. The TN count was highest for Insider (1841), indicating its ability to avoid false alarms;
- The overall ACC of the tools varied, ranging from 59.51% (Horusec) to 90.32% (Insider), showing differences in their effectiveness in correctly classifying vulnerabilities;
- FindSecBugs achieved the highest SEN at 96.08%, indicating its strong capability to identify true positives relative to other tools;
- PRE was the highest for Insider at 100.00%, suggesting that when it reported a positive result, it was almost always a true positive;
- The proportion of actual vulnerabilities detected by each tool varied, with FindSecBugs achieving 9.58%. However, some tools had much lower proportions of detected vulnerabilities, such as Graudit with 0.93%;
- The proportion of missed vulnerabilities also varied, with Graudit having the highest percentage at 9.05%;
- The proportion of FP varied, with FindSecBugs having a high FP rate of 37.51%;
- The proportion of correctly identified non-vulnerable instances (TN) was generally high for all tools, with Insider achieving the highest TN percentage at 90.02%.

Table 9 provides an analysis of the various SAST tool indicators for the Vulnerable App application. The results reveal important insights into each tool’s performance:

- FindSecBugs identified 24 TP, indicating its ability to uncover actual security issues within the application;
- Graudit detected 12 TP, suggesting a moderate capability to find vulnerabilities;
- Horusec found only four TPs, indicating limited effectiveness in identifying security issues;
- Insider identified just one TP, signifying a low capability for detecting vulnerabilities;
- ShiftLeftScan detected 10 TP, showing a moderate ability to identify security problems;
- Semgrep achieved the highest number of TPs, with 25, indicating a strong capability for uncovering vulnerabilities;
- FP was observed, with the highest count for Graudit (31) and the lowest for Insider (3). High FP counts can lead to resource-intensive investigations of false alarms;
- The tools correctly identified TNs, where no vulnerabilities were present. TN counts ranged from 26 (Graudit) to 55 (Insider), indicating the ability to avoid false alarms;
- The overall ACC of the tools varied, ranging from 39.18% (Graudit) to 76.29% (Semgrep), showing differences in their effectiveness in correctly classifying vulnerabilities;
- Semgrep achieved the highest SEN at 62.50%, indicating its strong capability to identify true positives relative to the other tools;
- PRE was the highest for Semgrep, at 75.76%, suggesting that when it reported a positive result, it was often a true positive;
- The proportion of actual vulnerabilities detected by each tool varied, with Semgrep achieving 25.77%. However, some tools had lower proportions of detected vulnerabilities, such as Horusec with 4.12%;
- The proportion of missed vulnerabilities FN varied, with Horusec having the highest percentage at 37.11%;
- The proportion of FP also varied, with Graudit having a relatively high FP rate of 31.96%;
- The proportion of correctly identified non-vulnerable instances (TN) was generally high for all tools, with Insider achieving the highest TN percentage, at 55.67%.

Table 10 provides average values for the selected SAST tool indicators for applications developed using Java technology. The results provide valuable insights into the performance of each tool:

- FindSecBugs had the highest average ACC, at 76.60%, indicating that it was generally effective in correctly classifying vulnerabilities for Java applications;
- Graudit had the lowest average ACC at 49.66%, suggesting that it had a lower overall accuracy compared to the other tools;
- FindSecBugs achieved the highest average SEN, at 85.23%, indicating its strong capability to identify true positives relative to the other tools;
- Insider had the lowest average SEN, at 2.88%, suggesting that it had difficulty in identifying actual security issues;
- PRE was the highest for FindSecBugs, at 59.85%, indicating that when it reported a positive result, it was often a true positive;
- Graudit had the lowest average PRE, at 13.43%, indicating a higher rate of false positives when it reported security issues;
- The proportion of TP among all vulnerabilities detected varied among the tools, with FindSecBugs achieving the highest average, at 24.07%;
- ShiftLeftScan had the lowest average TP percentage, at 13.16%, indicating a lower capability to identify true positives relative to the other tools;
- The proportion of FN varied among the tools, with Graudit having the highest average FN percentage, at 22.19%, indicating missed vulnerabilities;
- Insider had the lowest average FN percentage at 2.88%, suggesting that it had a lower tendency to miss vulnerabilities;
- FP was observed, with FindSecBugs having the highest average FP percentage, at 17.83%;
- Insider had the lowest average FP percentage, at 2.02%, indicating a lower rate of false alarms;
- The proportion of TN among all non-vulnerable instances varied among the tools, with Insider achieving the highest average TN percentage, at 68.34%;
- FindSecBugs had the lowest average TN percentage, at 52.53%, suggesting a higher rate of false alarms among non-vulnerable instances.

Based on the comprehensive analysis of various SAST tools applied to multiple Java applications, we can draw the following conclusions:

- FindSecBugs consistently outperformed the other tools in terms of ACC, SEN, and PRE across different Java applications. Graudit consistently performed the worst, with the lowest ACC, SEN, and PRE among the tools;
- ACC—FindSecBugs achieved the highest average ACC (76.60%), indicating its overall effectiveness in correctly classifying vulnerabilities for Java applications. Graudit had the lowest average ACC (49.66%), indicating its lower overall accuracy compared to the other tools;
- SEN—FindSecBugs had the highest average SEN (85.23%), demonstrating its strong capability to identify true positives relative to the other tools. Insider had the lowest average SEN (2.88%), indicating that it struggled to identify actual security issues effectively;
- PRE—FindSecBugs achieved the highest average PRE (59.85%), suggesting that when it reported a positive result, it was often a true positive. Graudit had the lowest average PRE (13.43%), indicating a higher rate of false positives when it reported security issues;
- TP and FN—FindSecBugs consistently identified a higher proportion of TP among all vulnerabilities detected, indicating its strong capability to find actual security issues. Graudit had the highest average FN percentage (22.19%), suggesting that it frequently missed vulnerabilities;
- FP and TN—Insider had the lowest average FP percentage (2.02%), indicating a lower rate of false alarms among non-vulnerable instances. FindSecBugs had the highest average FP percentage (17.83%), suggesting a higher rate of false alarms among non-vulnerable instances.

In summary, FindSecBugs was consistently the top-performing SAST tool, excelling in accuracy, sensitivity, and precision. It consistently achieved the highest proportion of true positives, while maintaining a reasonable accuracy. On the other hand, Graudit consistently performed the worst, with lower accuracy, sensitivity, and precision, and a higher rate of false negatives. The choice of SAST tool should consider the specific needs of the application and the importance of minimizing false alarms, while maximizing the detection of true security vulnerabilities.

### 5.2. Results for Applications Developed Using Javascript Technology

Table 11 presents the results of SAST tool evaluations for the Broken Crystals application. The results reveal important insights into each tool’s performance:

- Horusec detected the highest number of TPs, with 15, followed by Graudit with 9 and Semgrep with 6;
- Bearer had the highest number of FNs (25), indicating that it missed a significant portion of vulnerabilities. Semgrep and Horusec also had a substantial number of false negatives;
- Horusec reported the most FPs (104), potentially leading to unnecessary investigations, while Bearer had zero false positives;
- Semgrep achieved the highest accuracy at 85.26%, meaning it made fewer misclassifications. Horusec had the lowest accuracy, at 25.64%;
- Horusec demonstrated the highest sensitivity, at 55.56%, indicating its effectiveness in identifying true positives. Bearer had the lowest sensitivity at 7.41%, implying that it missed many vulnerabilities;
- Semgrep achieved the highest precision at 75.00%, meaning that when it reported a positive result, it was often accurate. Graudit had no precision because it reported no TPs.

Table 12 presents the results of SAST tool evaluations for the Damn Vulnerable Web Services application. The results reveal important insights into each tool’s performance:

- Semgrep performed the best in this category, with seven true positives, followed by Bearer with four and Horusec with three;
- Insider had the highest number of FNs (10), indicating it missed a significant portion of vulnerabilities. Other tools, such as Horusec and Graudit, also missed vulnerabilities;
- Bearer and Horusec both reported four false positives;
- Semgrep achieved the highest ACC, at 90.70%, indicating that it made fewer misclassifications. Graudit had the lowest accuracy, at 34.88%;
- Semgrep demonstrated the highest sensitivity, at 63.64%, indicating its effectiveness in identifying true positives. Insider had the lowest sensitivity at 9.09%, implying that it missed many vulnerabilities;
- Semgrep and Insider achieved the highest precision, at 100.00%. However, Insider reported a low number of vulnerabilities overall.

Table 13 presents the results of the assessment of SAST tools applied to the Juice Shop application. The results reveal important insights into each tool’s performance:

- Among the tools, Semgrep stands out with the highest number of TPs (20), indicating its effectiveness in identifying real vulnerabilities in the Juice Shop application. It was followed by Graudit, with 12 true positives;
- Insider had the highest number of false negatives (29), indicating that it failed to detect a significant number of vulnerabilities;
- Graudit reported the most FPs (76), followed by Horusec with 45.
- Bearer achieved the highest accuracy, at 84.21%, indicating that it made fewer misclassifications. Graudit had the lowest accuracy, at 36.84%, suggesting a higher likelihood of misidentifying vulnerabilities;
- Semgrep demonstrated the highest sensitivity at 62.50%, indicating its effectiveness in identifying true positives. Insider had the lowest sensitivity, at 9.38%, implying that it missed many vulnerabilities;
- Semgrep achieved the highest precision, at 60.61%. However, Graudit reported a high number of false positives, resulting in a low precision of 0.00%.

Table 14 presents the results of SAST tool evaluations for the NodeGoat application. The results reveal important insights into each tool’s performance:

- Semgrep and Bearer both achieved the highest TP count, with seven each;
- Insider and Horusec shared the highest FN count, with nine each;
- Graudit reported the most false positives, with 22;
- Semgrep achieved the highest accuracy, at 93.33%, indicating that it made fewer misclassifications;
- Semgrep had the highest sensitivity, at 63.64%;
- Semgrep also achieved the highest precision, at 87.50%.

Table 15 presents the average values for the selected indicators for SAST tools applied to applications developed using JavaScript technology. These averages provide an overview of the overall performance of each SAST tool across multiple applications:

- Bearer—On average, this SAST tool achieved an ACC% of 83.32%, SEN% of 33.10%, PRE% of 80.00%, TP% of 6.30%, FN% of 13.36%, and FP% of 3.33%. The TN% averaged at 77.02%;
- Graudit—The average performance of this tool included an accuracy of 51.68%, sensitivity of 31.34%, precision of 0.00%, true positive rate of 6.15%, false negative rate of 13.49%, and false positive rate of 34.83%. The true negative rate averaged at 45.52%;
- Horusec—On average, Horusec achieved an accuracy of 47.00%, sensitivity of 33.85%, precision of 15.78%, true positive rate of 6.62%, false negative rate of 13.03%, and false positive rate of 39.97%. The true negative rate averaged at 40.38%;
- Insider—The average performance of this tool resulted in an accuracy of 78.77%, sensitivity of 11.01%, precision of 51.39%, true positive rate of 2.06%, false negative rate of 17.59%, and false positive rate of 3.64%. The true negative rate averaged at 76.71%;
- Semgrep—On average, Semgrep achieved the highest accuracy of 88.21%, a sensitivity of 53.00%, precision of 80.78%, true positive rate of 10.65%, false negative rate of 9.00%, and false positive rate of 2.79%. The true negative rate averaged 77.56%.

These average values provide an overall picture of how each SAST tool performed when applied to JavaScript-based applications. Semgrep stands out, with a high accuracy, sensitivity, and precision, making it a strong choice for securing JavaScript applications. However, the selection of the most suitable tool should consider project-specific requirements and constraints;

Based on the comprehensive analysis of various SAST tools applied to multiple JavaScript applications, we can draw the following conclusions:

- The data highlight substantial variations in the performance of the different SAST tools when applied to JavaScript technology. Each tool demonstrated varying degrees of accuracy, sensitivity, precision, and other indicators;
- Semgrep consistently stood out as the most accurate tool, with an average accuracy of 88.21%, making it a strong candidate for JavaScript applications;
- Semgrep and Bearer exhibited higher sensitivities, indicating their effectiveness in identifying security vulnerabilities;
- Semgrep and Bearer also excelled in precision. Reducing false positives is crucial to minimize the time spent investigating non-existent vulnerabilities;
- Semgrep consistently maintained a high true positive rate.
- Horusec showed promise in precision but lagged in sensitivity, which might be suitable for certain scenarios.

In conclusion, the choice of a SAST tool for JavaScript applications should be made based on a careful evaluation of the specific requirements and constraints of the project. While Semgrep consistently exhibited a strong overall performance, other tools may excel in particular areas or be better suited for specific use cases. A comprehensive security strategy should involve the selection of the right tools, continuous monitoring, and expert analysis, to ensure robust protection against vulnerabilities in JavaScript-based applications.

### 5.3. Results for Applications Developed Using PHP Technology

Table 16 presents the results of the assessment of SAST tools applied to the Conviso Vulnerable Web application. The results reveal important insights into each tool’s performance:

- Horusec stands out with a 100% sensitivity, indicating that it successfully identified all true positives. However, it is essential to consider the balance between sensitivity and specificity, as achieving 100% sensitivity might lead to a high number of false positives;
- Graudit and Horusec demonstrated a perfect precision, with 100%, meaning that all reported vulnerabilities were true positives. Conversely, ShiftLeft Scan and Semgrep showed 0% precision, implying that they reported only false positives in this context;
- Graudit, Horusec, PHP_CS, and Progpilot exhibited true positive rates ranging from 20% to 44.44%, while ShiftLeft Scan and Semgrep had 0% true positive rates, indicating that they failed to identify any true positives;
- Semgrep had a notably high false positive rate of 90%, which means it reported many issues that were not actual vulnerabilities in the application;
- Some tools, such as Horusec and Progpilot, reported true negatives, indicating that they correctly identified non-vulnerable portions of the application;
- Horusec achieved 100% accuracy, which is commendable. However, it is crucial to consider accuracy in conjunction with other metrics, as a high accuracy rate may be achieved by reporting fewer vulnerabilities, potentially missing real issues.

Table 17 presents the results of an assessment of SAST tools applied to the Damn Vulnerable Web application. The results reveal important insights into each tool’s performance:

- Horusec stands out, with a high sensitivity (90.40%), indicating that it successfully identified a substantial portion of true positives. Conversely, Progpilot showed a sensitivity of only 12.80%, suggesting it missed many true positives;
- Progpilot demonstrated a 100% precision, implying that all reported vulnerabilities were true positives. However, ShiftLeft Scan had a relatively low precision, at 55.26%, indicating a higher likelihood of false positives;
- Horusec had a high true positive rate (25.17%), while Progpilot and Semgrep had lower rates, implying they missed a significant number of true positives;
- Horusec and PHP_CS had relatively high FP rates, indicating they reported some issues that were not actual vulnerabilities in the application. Semgrep had the lowest FP rate among the tools;
- Some tools, such as Graudit, PHP_CS, and ShiftLeft Scan, reported TNs, indicating that they correctly identified non-vulnerable portions of the application;
- Graudit, Progpilot, and ShiftLeft Scan exhibited reasonably high accuracy rates. However, it is essential to consider accuracy in conjunction with other metrics, to assess the overall performance of each tool.

Table 18 presents the results of the assessment of SAST tools applied to the WackoPicko application. The results reveal important insights into each tool’s performance:

- Horusec stands out with a high sensitivity (93.40%), indicating that it successfully identified a substantial portion of true positives. Conversely, ShiftLeft Scan and Semgrep had much lower sensitivities, implying they missed many true positives;
- Progpilot demonstrated the highest precision, at 92.00%, implying that the vulnerabilities it reported were highly likely to be true positives. Other tools, such as Graudit and Horusec, had a lower precision;
- Horusec and Graudit exhibited reasonably high true positive rates. In contrast, Semgrep and ShiftLeft Scan had much lower rates, indicating they missed a significant number of true positives;
- Horusec and Progpilot had relatively low false positive rates, indicating that they reported fewer false alarms. ShiftLeft Scan and Semgrep had slightly higher false positive rates;
- Most tools reported true negatives, indicating that they correctly identified non-vulnerable portions of the application;
- Horusec demonstrated the highest accuracy at 68.00%, followed closely by Progpilot at 71.56%. Semgrep had the lowest accuracy among the tools.

Table 19 presents the results of the assessment of SAST tools applied to the Xtreme Vulnerable Web application. The results reveal important insights into each tool’s performance:

- Horusec exhibited a high sensitivity (90.20%), indicating its ability to detect a substantial number of true positives. Progpilot also showed good sensitivity (39.22%). On the other hand, ShiftLeft Scan and Semgrep had lower sensitivity values;
- Progpilot demonstrated the highest precision at 86.96%, indicating that the vulnerabilities it reported were highly likely to be true positives. Horusec had a notably lower precision;
- Horusec and Progpilot exhibited reasonable true positive rates. In contrast, Semgrep and ShiftLeft Scan had lower rates, implying that they missed a significant number of true positives;
- Horusec and ShiftLeft Scan reported a high number of false positives, while Progpilot and Semgrep had lower false positive rates;
- Most tools correctly identified true negatives, which are non-vulnerable portions of the application;
- Graudit and Progpilot demonstrated a high accuracy, with Progpilot being the most accurate, at 91.15%. Horusec had a notably lower accuracy score.

Table 20 presents the average values of the selected indicators for SAST tools applied to applications developed using PHP technology. These averages provide an overview of the overall performance of each SAST tool across multiple applications:

- Among the SAST tools, Progpilot stands out with the highest average accuracy, at 72.11%, indicating its ability to correctly classify vulnerabilities and non-vulnerable code. Graudit and ShiftLeft Scan also exhibited relatively high accuracies, while Horusec, PHP_CS, and Semgrep had lower average accuracy scores;
- Horusec demonstrated the highest average sensitivity, at 93.50%, suggesting that it excelled in identifying true positives, although this was balanced by other factors. Graudit also had a decent sensitivity score. On the other hand, Semgrep had a notably lower average sensitivity;
- Progpilot stands out with the highest average precision score, at 94.74%, indicating that the vulnerabilities it reported were highly likely to be true positives. ShiftLeft Scan and Graudit also showed a good average precision. Semgrep had the lowest average precision;
- Progpilot and Horusec exhibited reasonable average true positive rates, which indicatd their effectiveness in identifying actual vulnerabilities. Semgrep had the lowest average TP rate;
- Semgrep and ShiftLeft Scan had the highest average false negative rates, suggesting that they missed a substantial number of vulnerabilities in PHP applications. Horusec had the lowest average FN rate;
- Horusec reported a high average false positive rate, indicating that it identified vulnerabilities that were not actually present. In contrast, Progpilot and Semgrep had the lowest average FP rates;
- Progpilot achieved the highest average true negative rate, suggesting that it effectively identified non-vulnerable portions of the code. Semgrep and ShiftLeft Scan also exhibited good average TN rates.

In conclusion, the choice of SAST tool for PHP applications should consider a balance between accuracy, sensitivity, and precision. Progpilot excels in precision but may miss some vulnerabilities. Horusec has high sensitivity but reports more false positives. Graudit and ShiftLeft Scan offer a good trade-off between these metrics. Semgrep demonstrated a lower overall performance, particularly in sensitivity and precision. The selection should align with the specific requirements and constraints of the project, and fine-tuning may be necessary for comprehensive security testing.

### 5.4. Results for Applications Developed Using Python Technology

Table 21 presents the results of the assessment of SAST tools applied to the Damn Small Vulnerable Web application. The results reveal important insights into each tool’s performance:

- Aura achieved a SEN of 38.89%, indicating its ability to detect TPs, but with some FNs. The PRE was 100.00%, meaning it reported no FPs. The ACC was 47.62%;
- Graudit showed limited sensitivity (0.00%), with no true positives and limited precision (0.00%) as it did not report any true positives. The accuracy was 14.29%,
- Horusec demonstrated a high precision (85.71%) but moderate sensitivity (66.67%), indicating a trade-off between false positives and false negatives. The accuracy was 61.90%;
- Bandit had a precision of 84.62%, suggesting a low rate of false positives, and a sensitivity of 61.11%. The accuracy was 57.14%;
- ShiftLeft Scan achieved a perfect precision of 100.00%, reporting no false positives. However, the sensitivity was quite low at 5.56%, resulting in a trade-off between precision and sensitivity. The accuracy was 19.05%;
- Semgrep exhibited a high precision (90.00%) and a balanced sensitivity (50.00%). The accuracy was 52.38%.

In summary, the SAST tools exhibited varying performance for the Damn Small Vulnerable Web application. Aura achieved perfect precision but had a limited sensitivity. Horusec balanced precision and sensitivity, while Graudit showed limited performance. Bandit performed well, with high precision and sensitivity. ShiftLeft Scan excelled in precision but had limited sensitivity. Semgrep achieved a good balance between precision and sensitivity.

Table 22 presents the results of the assessment of SAST tools applied to the Damn Vulnerable GraphQL application. The results reveal important insights into each tool’s performance:

- Aura achieved an ACC of 36.36%. It had a SEN of 33.33% and a precision PRE of 75.00%;
- Graudit exhibited limited performance, with an accuracy of 18.18%. It reported no TPs and had a SEN and PRE of 0.00%.
- Horusec achieved an accuracy of 27.27%. It had a SEN of 33.33% and a PRE of 60.00%.
- Bandit also had an accuracy of 27.27%. It had a SEN and PRE of 33.33% and 60.00%, respectively;
- ShiftLeft Scan demonstrated the highest accuracy, at 63.64%. It had a SEN of 55.56% and a perfect precision of 100.00%;
- Semgrep had an accuracy of 45.45%. It achieved a balanced SEN and PRE of 33.33% and 100.00%, respectively.

In summary, the SAST tools exhibited varying levels of performance when analyzing the Damn Vulnerable GraphQL Application. ShiftLeft Scan stood out, with the highest accuracy and perfect precision, indicating a low rate of false positives. However, it also reported a relatively higher number of false negatives. Semgrep achieved a balanced performance, with good precision and sensitivity. Other tools, such as Aura, Horusec, and Bandit, showed moderate performance with different trade-offs between accuracy, precision, and sensitivity. Graudit had a limited performance, with no true positives reported.

Table 23 presents the results of the assessment of SAST tools applied to the Damn Vulnerable Python Web application. The results reveal important insights into each tool’s performance:

- Aura reported 1 TP and 2 FN. It achieved an ACC of 71.43%, a SEN of 33.33%, and a perfect PRE of 100.00%;
- Graudit reported no TPs and three FNs. It had an ACC of 57.14%, a SEN of 0.00%, and a PRE of 0.00%;
- Horusec achieved two TPs and one FN. It had the highest ACC at 85.71%, a SEN of 66.67%, and a perfect PRE of 100.00%;
- Bandit reported one TP and two FNs. It had an ACC of 71.43%, a SEN of 33.33%, and a PRE of 100.00%;
- ShiftLeft Scan reported one TP and two FNs. It had the lowest ACC, at 14.29%, a SEN of 33.33%, and a PRE of 20.00%;
- Semgrep reported two TPs and one FN. It achieved an ACC of 85.71%, a SEN of 66.67%, and a perfect PRE of 100.00%.

In summary, the SAST tools provided varying results when analyzing the Damn Vulnerable Python Web Application. Horusec and Semgrep demonstrated the highest accuracy and precision, indicating their ability to identify vulnerabilities, with fewer false positives. Aura and Bandit showed moderate performance, with a balance between accuracy, sensitivity, and precision. Graudit reported limited performance, with no true positives, while ShiftLeft Scan had the lowest accuracy and precision among the tools. The choice of a specific tool should consider the trade-offs between accuracy and precision, depending on the specific application’s security requirements.

Table 24 presents the results of an assessment of SAST tools applied to the Tiredful API application. The results reveal important insights into each tool’s performance:

- Aura reported two TPs and six FNs. It achieved an ACC of 37.50%, a SEN of 25.00%, and a PRE of 33.33%;
- Graudit reported no TPs and eight FNs. It had an ACC of 50.00%, a SEN of 0.00%, and a PRE of 0.00%;
- Horusec achieved two TPs and six FNs. It had an ACC of 62.50%, a SEN of 25.00%, and a perfect PRE of 100.00%;
- Bandit reported two TPs and six FNs. It had an ACC of 62.50%, a SEN of 25.00%, and a perfect PRE of 100.00%. ShiftLeft Scan reported six TPs and two FNs. It had an ACC of 62.50%, a SEN of 75.00%, and a PRE of 60.00%;
- Semgrep reported three TPs and five FNs. It achieved an ACC of 68.75%, a SEN of 37.50%, and a perfect PRE of 100.00%.

In summary, the SAST tools provided varying results when analyzing the Tiredful API application. Semgrep demonstrated the highest accuracy, sensitivity, and precision, indicating its ability to identify vulnerabilities effectively. Horusec and Bandit showed moderate performance, with a balanced accuracy and precision. Aura had the lowest accuracy and precision among the tools. The choice of a specific tool should consider the trade-offs between accuracy and precision, depending on the specific application’s security requirements.

Table 25 presents the average values of selected SAST tool indicators for applications developed using Python technology. These averages provide an overview of the overall performance of each SAST tool across multiple applications:

- On average, the SAST tools achieved accuracy scores ranging from approximately 34.90% to 63.07%. Semgrep had the highest average accuracy, indicating that it provided the most correct results on average. This suggests that Semgrep can be relied upon to accurately identify vulnerabilities in Python code;
- The average SEN scores ranged from around 0.00% to 47.92%. Semgrep and Horusec exhibited relatively better sensitivity, making them suitable for detecting a broad range of vulnerabilities;
- The PRE scores varied widely, with Semgrep achieving the highest average precision (97.50%). This means that when Semgrep flagged a vulnerability, it was highly likely to be a true positive;
- Semgrep had the highest average TP rate (29.36%), suggesting that it had a reasonably good ability to find vulnerabilities within Python applications;
- The average FN rates ranged from approximately 12.50% to 65.10%. Graudit had the highest FN rate, implying that it missed a substantial number of vulnerabilities. Semgrep and ShiftLeft Scan demonstrated relatively lower FN rates;
- The average FP rates ranged from around 0.00% to 20.54%. ShiftLeft Scan had the highest FP rate, followed by Aura. Semgrep produced the fewest false alarms;
- Graudit achieved the highest TN rate, followed by Semgrep.

In summary, Semgrep consistently performed well across multiple evaluation criteria, making it a strong candidate for analyzing Python applications for security vulnerabilities. However, the choice of the most suitable SAST tool should also consider project-specific requirements; the types of vulnerabilities you are targeting; and trade-offs between accuracy, sensitivity, and precision. Additionally, it is essential to keep in mind that the effectiveness of these tools can vary depending on the specific codebase and the complexity of the application. Therefore, conducting comprehensive testing and fine-tuning the tool’s configurations may be necessary to achieve optimal results.

### 5.5. Scan Duration

Table 26 presents a comparison of the scan duration times (in seconds) for the various security scanning tools across different applications. The results are given rounded to the nearest second. The times were rounded up. The table demonstrates a significant variability in scan duration times across different security scanning tools and applications. Scan times ranged from a few seconds to several minutes, depending on the combination of the tool and the target application. Bandit consistently demonstrated fast scan times, typically taking only 1 s to complete its analysis, regardless of the application. Other tools, such as Graudit, also exhibited fast scan times, completing scans in just 1 s for most applications. The choice of the target application had a considerable impact on scan duration. Some applications, such as Broken Crystals, required longer scan times, with Bearer CLI taking 510 s for this particular application. Semgrep and ShiftLeft Scan both showed competitive scan times across a wide range of applications. They tended to provide relatively quick results, without compromising on scan depth. On average, across all applications, Bearer CLI had the longest scan time, averaging 181 s (approximately 3 min). In contrast, Bandit, Graudit, and Semgrep had average scan times of 1 s. While some tools, such as Bandit, consistently exhibited fast scan times, they might have limitations in terms of the types of vulnerabilities they can detect. Therefore, the choice of a tool should consider not only scan duration but also the tool’s coverage and effectiveness in identifying vulnerabilities. Scan times can also be influenced by tool configurations, such as the scan depth and the number of rules enabled. Adjusting these configurations might help balance scan duration with the depth of analysis. The complexity and size of the target application play a significant role in scan times. For example, Bearer CLI takes longer to scan more complex applications, while smaller applications generally have shorter scan times. In practice, organizations should consider a balance between scan duration and the tool’s ability to identify vulnerabilities effectively. A tool with a very fast scan time but low detection rates may not be as valuable as a slightly slower tool with more comprehensive coverage. Additionally, organizations may need to factor in their specific requirements, such as the need for quick feedback in a continuous integration/continuous deployment (CI/CD) pipeline or the depth of analysis required for critical applications. Overall, the choice of a security scanning tool should be based on a combination of factors, including scan duration, effectiveness, coverage, and the specific needs of the project or organization.

## 6. Conclusions

The primary objective of this study was to conduct a comprehensive comparative analysis of open-source static code analysis tools, with a specific focus on their efficacy in identifying vulnerabilities. The investigation hinged on the examination of the vulnerabilities cataloged by these tools and their subsequent application in scrutinizing vulnerable web applications crafted in selected programming languages.

To facilitate a testing environment, a dedicated test infrastructure was established. This infrastructure encompassed a host machine and a virtual machine, serving as the platform for experimental execution. The study provided concise descriptions of the scrutinized tools and the web applications subjected to evaluation. A total of eleven distinct tools, each tailored to diverse technologies prevalent in web application development, underwent assessment. The research encompassed a broad spectrum of programming languages, including Java, JavaScript, PHP, and Python, and involved the analysis of sixteen vulnerable web applications. The analysis adhered to a structured methodology, where scan reports were standardized into a uniform format, outcomes for each application were consolidated, and each detected vulnerability was categorized into one of three labels: True Positives (TP), False Positives (FP), or Not Applicable (N/A). Vulnerabilities designated as N/A were excluded from subsequent analyses. Finally, performance metrics were computed for each tool, and the results underwent meticulous scrutiny.

The findings emerging from this exhaustive analysis of security testing tools for static code analysis underscore a pivotal realization: the absence of a universally impeccable tool. A salient example is Semgrep, which exhibited outstanding performance when evaluating applications developed using JavaScript technologies but faltered when confronted with applications forged in PHP technologies. This observation underscores the intricacy of tool selection, as distinct tools exhibit superior efficacy in disparate contexts. For instance, native tools specifically engineered for particular technologies, such as Java and PHP, generally outperformed their counterparts when evaluated within their respective domains. Conversely, “multitechnology” tools demonstrated enhanced effectiveness when scrutinizing applications developed with JavaScript and Python technologies.

Furthermore, it is imperative to emphasize that the deliberate inclusion of security vulnerabilities in the test applications amplifies the real-world relevance of this study’s outcomes. These insights transcend the domain of web applications, as the tested tools are inherently versatile and can be applied to a spectrum of application types, including those designed for embedded systems, IoT, or sensor-equipped devices. This versatility accentuates their relevance in fortifying overall software security across diverse domains, extending beyond the confines of web development.

In summation, this study advocates for a nuanced approach to tool selection in the realm of static code analysis, given the absence of a universally flawless tool. Tailoring tool choices to the specific technologies in use emerged as a critical consideration for effective vulnerability detection. The deliberate inclusion of security errors in the test applications reinforces the practical applicability of the study’s findings, thereby elucidating the versatility of these tools in diverse application landscapes beyond web development.

## Figures and Tables

**Table 1:** Used hardware and software.

Asset	Type	Version/Data
AMD Ryzen 5 3600	CPU	6 physical/12 logical cores, 3.6 GHz (4.2 GHz turbo)
Kingston KF3200C16D4/16GX	RAM	32 GB, DDR4-3200/PC4-25600
Crucial CT525MX300SSD1	SSD	read 530 MB/s, write 510 MB/s
Microsoft Windows Home	Host operating system	22 621.1555
Oracle VM VirtualBox	Virtualization technology	7.0.8 r156879
Ubuntu Server	Virtualized operating system	22.04.2 LTS

**Table 2:** Used web applications.

Application	Technology	Version from the Date
EasyBuggy	Java	2 December 2021
Java Vulnerable Lab	Java	24 January 2019
SasanLabs VulnerableApp	Java	14 August 2022
Security Shepherd	Java	24 October 2018
Broken Crystals	JavaScript	4 May 2023
Damn Vulnerable Web Services	JavaScript	12 February 2023
OWASP Juice Shop	JavaScript	4 August 2019
OWASP NodeGoat	JavaScript	4 August 2019
Conviso Vulnerable Web Application	PHP	28 April 2023
Damn Vulnerable Web Application	PHP	4 May 2023
WackoPicko	PHP	17 November 2021
Xtreme Vulnerable Web Application	PHP	12 September 2020
Damn Small Vulnerable Web	Python	22 July 2021
Damn Vulnerable GraphQL Application	Python	25 April 2023
Damn Vulnerable Python Web Application	Python	4 November 2022
Tiredful API	Python	7 September 2020

**Table 3:** Tested security code analysis tools.

Name	Version
Bearer CLI	1.5.1
Floe phpcs-security-audit	2.0.1
Graudit	3.5
InsiderSec Insider CLI	3.0.0
OWASP FindSecBugs	1.12.0
Progpilot	1.0.2
PyCQA Bandit	1.7.5
Semgrep	1.20.0
ShiftLeft Inc. Scan	2.1.2
SourceCode.AI Aura	2.1
Zup Horusec	2.8.0

**Table 4:** Support of SAST tools for individual technologies.

Tool	Python	Java	JavaScript	PHP
Bearer CLI	No	No	Yes	No
Floe phpcs-security-audit	No	No	No	Yes
Graudit	Yes	Yes	Yes	Yes
InsiderSec Insider CLI	No	Yes	Yes	No
OWASP FindSecBugs	No	Yes	No	No
Progpilot	No	No	No	Yes
PyCQA Bandit	Yes	No	No	No
Semgrep	Yes	Yes	Yes	Yes
ShiftLeft Inc. Scan	Yes	Yes	No	Yes
SourceCode.AI Aura	Yes	No	No	No
Zup Horusec	Yes	Yes	Yes	Yes

**Table 5:** Matrix of conducted experiments.

Application	Aura	Bandit	Bearer CLI	FindSecBugs	Graudit	Horusec	Insider CLI	Phpcs-Security-Audit	Progpilot	ShiftLeft Scan	Semgrep
EasyBuggy	N	N	N	Y	Y	Y	Y	N	N	Y	Y
Java Vulnerable Lab	N	N	N	Y	Y	Y	Y	N	N	Y	Y
Security Shepherd	N	N	N	Y	Y	Y	Y	N	N	Y	Y
Vulnerable App	N	N	N	Y	Y	Y	Y	N	N	Y	Y
Broken Crystals	N	N	Y	N	Y	Y	Y	N	N	N	Y
Damn Vulnerable Web Services	N	N	Y	N	Y	Y	Y	N	N	N	Y
Juice Shop	N	N	Y	N	Y	Y	Y	N	N	N	Y
NodeGoat	N	N	Y	N	Y	Y	Y	N	N	N	Y
Conviso Vulnerable Web Application	N	N	N	N	Y	Y	N	Y	Y	Y	Y
Damn Vulnerable Web Application	N	N	N	N	Y	Y	N	Y	Y	Y	Y
WackoPicko	N	N	N	N	Y	Y	N	Y	Y	Y	Y
Xtreme Vulnerable Web Application	N	N	N	N	Y	Y	N	Y	Y	Y	Y
Damn Small Vulnerable Web	Y	Y	N	N	Y	Y	N	N	N	Y	Y
Damn Vulnerable GraphQL Application	Y	Y	N	N	Y	Y	N	N	N	Y	Y
Damn Vulnerable Python Web Application	Y	Y	N	N	Y	Y	N	N	N	Y	Y
Tiredful API	Y	Y	N	N	Y	Y	N	N	N	Y	Y

**Table 6:** The values of SAST tool indicators for the EasyBuggy application.

	FindSecBugs	Graudit	Horusec	Insider	ShiftLeftScan	Semgrep
**TP**	29	0	2	1	13	8
**FN**	4	33	31	32	20	25
**FP**	15	0	38	2	6	3
**TN**	41	56	18	54	50	53
**P**	33					
**N**	56					
**TOTAL**	89					
**ACC [%]**	78.65	62.92	22.47	61.80	70.79	68.54
**SEN [%]**	87.88	0.00	6.06	3.03	39.39	24.24
**PRE [%]**	65.91	0.00	5.00	33.33	68.42	72.73
**TP [%]**	32.58	0.00	2.25	1.12	14.61	8.99
**FN [%]**	4.49	37.08	34.83	35.96	22.47	28.09
**FP [%]**	16.85	0.00	42.70	2.25	6.74	3.37
**TN [%]**	46.07	62.92	20.22	60.67	56.18	59.55

**Table 7:** The values of SAST tool indicators for the Java Vulnerable Lab application.

	FindSecBugs	Graudit	Horusec	Insider	ShiftLeftScan	Semgrep
**TP**	32	18	5	1	21	14
**FN**	1	15	28	32	12	19
**FP**	5	70	3	3	3	4
**TN**	71	6	73	73	73	72
**P**	33					
**N**	76					
**TOTAL**	109					
**ACC [%]**	94.50	22.02	71.56	67.89	86.24	78.90
**SEN [%]**	96.97	54.55	15.15	3.03	63.64	42.42
**PRE [%]**	86.49	20.45	62.50	25.00	87.50	77.78
**TP [%]**	29.36	16.51	4.59	0.92	19.27	12.84
**FN [%]**	0.92	13.76	25.69	29.36	11.01	17.43
**FP [%]**	4.59	64.22	2.75	2.75	2.75	3.67
**TN [%]**	65.14	5.50	66.97	66.97	66.97	66.06

**Table 8:** The values of SAST tool indicators for the Security Shepherd application.

	FindSecBugs	Graudit	Horusec	Insider	ShiftLeftScan	Semgrep
**TP**	196	19	177	6	173	28
**FN**	8	185	27	198	31	176
**FP**	767	336	801	0	149	364
**TN**	1074	1505	1040	1841	1692	1477
**P**	204					
**N**	1841					
**TOTAL**	2045					
**ACC [%]**	62.10	74.52	59.51	90.32	91.20	73.59
**SEN [%]**	96.08	9.31	86.76	2.94	84.80	13.73
**PRE [%]**	20.35	5.35	18.10	100.00	53.73	7.14
**TP [%]**	9.58	0.93	8.66	0.29	8.46	1.37
**FN [%]**	0.39	9.05	1.32	9.68	1.52	8.61
**FP [%]**	37.51	16.43	39.17	0.00	7.29	17.80
**TN [%]**	52.52	73.59	50.86	90.02	82.74	72.22

**Table 9:** The values of SAST tool indicators for the Vulnerable App application.

	FindSecBugs	Graudit	Horusec	Insider	ShiftLeftScan	Semgrep
**TP**	24	12	4	1	10	25
**FN**	16	28	36	39	30	15
**FP**	12	31	12	3	9	8
**TN**	45	26	45	54	48	49
**P**	40					
**N**	57					
**TOTAL**	97					
**ACC [%]**	71.13	39.18	50.52	56.70	59.79	76.29
**SEN [%]**	60.00	30.00	10.00	2.50	25.00	62.50
**PRE [%]**	66.67	27.91	25.00	25.00	52.63	75.76
**TP [%]**	24.74	12.37	4.12	1.03	10.31	25.77
**FN [%]**	16.49	28.87	37.11	40.21	30.93	15.46
**FP [%]**	12.37	31.96	12.37	3.09	9.28	8.25
**TN [%]**	46.39	26.80	46.39	55.67	49.48	50.52

**Table 10:** Average values of the selected SAST tool indicators for applications developed using Java technology.

	FindSecBugs	Graudit	Horusec	Insider	ShiftLeftScan	Semgrep
**ACC [%]**	76.60	49.66	51.01	69.18	77.00	74.33
**SEN [%]**	85.23	23.46	29.49	2.88	53.21	35.72
**PRE [%]**	59.85	13.43	27.65	45.83	65.57	58.35
**TP [%]**	24.07	7.45	4.90	0.84	13.16	12.24
**FN [%]**	5.57	22.19	24.74	27.80	16.48	17.40
**FP [%]**	17.83	28.15	24.25	2.02	6.51	8.27
**TN [%]**	52.53	42.21	46.11	68.34	63.84	62.09

**Table 11:** The values of SAST tool indicators for the Broken Crystals application.

	Bearer	Graudit	Horusec	Insider	Semgrep
**TP**	2	9	15	2	6
**FN**	25	18	12	25	21
**FP**	0	21	104	2	2
**TN**	129	108	25	127	127
**P**	27				
**N**	129				
**TOTAL**	156				
**ACC [%]**	83.97	75.00	25.64	82.69	85.26
**SEN [%]**	7.41	33.33	55.56	7.41	22.22
**PRE [%]**	100.00	0.00	12.61	50.00	75.00
**TP [%]**	1.28	5.77	9.62	1.28	3.85
**FN [%]**	16.03	11.54	7.69	16.03	13.46
**FP [%]**	0.00	13.46	66.67	1.28	1.28
**TN [%]**	82.69	69.23	16.03	81.41	81.41

**Table 12:** Values of the SAST tool indicators for the Damn Vulnerable Web Services application.

	Bearer	Graudit	Horusec	Insider	Semgrep
**TP**	4	3	3	1	7
**FN**	7	8	8	10	4
**FP**	4	20	9	0	0
**TN**	28	12	23	32	32
**P**	11				
**N**	32				
**TOTAL**	43				
**ACC [%]**	74.42	34.88	60.47	76.74	90.70
**SEN [%]**	36.36	27.27	27.27	9.09	63.64
**PRE [%]**	50.00	00.00	25.00	100.00	100.00
**TP [%]**	9.30	6.98	6.98	2.33	16.28
**FN [%]**	16.28	18.60	18.60	23.26	9.30
**FP [%]**	9.30	46.51	20.93	0.00	0.00
**TN [%]**	65.12	27.91	53.49	74.42	74.42

**Table 13:** The values of SAST tool indicators for the Juice Shop application.

	Bearer	Graudit	Horusec	Insider	Semgrep
**TP**	8	12	11	3	20
**FN**	24	20	21	29	12
**FP**	0	76	45	6	13
**TN**	120	44	75	114	107
**P**	32				
**N**	120				
**TOTAL**	152				
**ACC [%]**	84.21	36.84	56.58	76.97	83.55
**SEN [%]**	25.00	37.50	34.38	9.38	62.50
**PRE [%]**	100.00	0.00	19.64	33.33	60.61
**TP [%]**	5.26	7.89	7.24	1.97	13.16
**FN [%]**	15.79	13.16	13.82	19.08	7.89
**FP [%]**	0.00	50.00	29.61	3.95	8.55
**TN [%]**	78.95	28.95	49.34	75.00	70.39

**Table 14:** The values of SAST tool indicators for the NodeGoat application.

	Bearer	Graudit	Horusec	Insider	Semgrep
**TP**	7	3	2	2	7
**FN**	4	8	9	9	4
**FP**	3	22	32	7	1
**TN**	61	42	32	57	63
**P**	32				
**N**	120				
**TOTAL**	152				
**ACC [%]**	90.67	60.00	45.33	78.67	93.33
**SEN [%]**	63.64	27.27	18.18	18.18	63.64
**PRE [%]**	70.00	0.00	5.88	22.22	87.50
**TP [%]**	9.33	4.00	2.67	2.67	9.33
**FN [%]**	5.33	10.67	12.00	12.00	5.33
**FP [%]**	4.00	29.33	42.67	9.33	1.33
**TN [%]**	81.33	56.00	42.67	76.00	84.00

**Table 15:** Average values of selected SAST tool indicators for applications developed using JavaScript technology.

	Bearer	Graudit	Horusec	Insider	Semgrep
**ACC [%]**	83.32	51.68	47.00	78.77	88.21
**SEN [%]**	33.10	31.34	33.85	11.01	53.00
**PRE [%]**	80.00	0.00	15.78	51.39	80.78
**TP [%]**	6.30	6.15	6.62	2.06	10.65
**FN [%]**	13.36	13.49	13.03	17.59	9.00
**FP [%]**	3.33	34.83	39.97	3.64	2.79
**TN [%]**	77.02	45.52	40.38	76.71	77.56

**Table 16:** The values of the SAST tool indicators for the Conviso Vulnerable Web application.

	Graudit	Horusec	PHP_CS	Progpilot	ShiftLeft Scan	Semgrep
**TP**	4	9	2	4	2	0
**FN**	5	0	7	5	7	9
**FP**	1	0	0	0	0	0
**TN**	0	1	1	1	1	1
**P**	9					
**N**	1					
**TOTAL**	10					
**ACC [%]**	40.00	100.00	30.00	50.00	30.00	10.00
**SEN [%]**	44.44	100.00	22.22	44.44	22.22	0.00
**PRE [%]**	80.00	100.00	100.00	100.00	100.00	0.00
**TP [%]**	40.00	90.00	20.00	40.00	20.00	0.00
**FN [%]**	40.00	90.00	20.00	40.00	20.00	0.00
**FP [%]**	50.00	0.00	70.00	50.00	70.00	90.00
**TN [%]**	0.00	10.00	10.00	10.00	10.00	10.00

**Table 17:** The values of the SAST tool indicators for the Damn Vulnerable Web application.

	Graudit	Horusec	PHP_CS	Progpilot	ShiftLeft Scan	Semgrep
**TP**	4	9	2	4	2	0
**FN**	5	0	7	5	7	9
**FP**	1	0	0	0	0	0
**TN**	0	1	1	1	1	1
**P**	125					
**N**	324					
**TOTAL**	449					
**ACC [%]**	63.93	35.63	67.04	75.72	73.94	74.61
**SEN [%]**	42.40	90.40	32.80	12.80	33.60	15.20
**PRE [%]**	37.06	28.97	39.05	100.00	55.26	70.37
**TP [%]**	11.80	25.17	9.13	3.35	9.35	4.23
**FN [%]**	16.04	2.67	18.71	24.28	18.49	23.61
**FP [%]**	20.04	61.69	14.25	0.00	7.57	1.78
**TN [%]**	52.12	10.47	57.91	72.16	64.59	70.38

**Table 18:** The values of the SAST tool indicators for the WackoPicko application.

	Graudit	Horusec	PHP_CS	Progpilot	ShiftLeft Scan	Semgrep
**TP**	62	99	62	46	11	7
**FN**	44	7	44	60	95	99
**FP**	28	113	58	4	1	2
**TN**	91	6	61	115	118	117
**P**	106					
**N**	119					
**TOTAL**	225					
**ACC [%]**	68.00	46.67	54.67	71.56	57.33	55.11
**SEN [%]**	58.49	93.40	58.49	43.40	10.38	6.60
**PRE [%]**	68.89	46.70	51.67	92.00	91.67	77.78
**TP [%]**	27.56	44.00	27.56	20.44	4.89	3.11
**FN [%]**	19.56	3.11	19.56	26.67	42.22	44.00
**FP [%]**	12.44	50.22	25.78	1.78	0.44	0.89
**TN [%]**	40.44	2.67	27.11	51.11	52.44	52.00

**Table 19:** The values of SAST tool indicators for the Xtreme Vulnerable Web application.

	Graudit	Horusec	PHP_CS	Progpilot	ShiftLeft Scan	Semgrep
**TP**	18	46	17	20	9	8
**FN**	33	5	34	31	42	43
**FP**	9	325	302	3	5	6
**TN**	324	8	31	330	328	327
**P**	51					
**N**	333					
**TOTAL**	384					
**ACC [%]**	89.06	14.06	12.50	91.15	87.76	87.24
**SEN [%]**	35.29	90.20	33.33	39.22	17.65	15.69
**PRE [%]**	66.67	12.40	5.33	86.96	64.29	57.14
**TP [%]**	4.69	11.98	4.43	5.21	2.34	2.08
**FN [%]**	8.59	1.30	8.85	8.07	10.94	11.20
**FP [%]**	2.34	84.64	78.65	0.78	1.30	1.56
**TN [%]**	84.38	2.08	8.07	85.94	85.42	85.16

**Table 20:** Average values of the selected SAST tool indicators for applications developed using PHP technology.

	Graudit	Horusec	PHP_CS	Progpilot	ShiftLeft Scan	Semgrep
**ACC [%]**	65.25	49.09	41.05	72.11	62.26	56.74
**SEN [%]**	45.16	93.50	36.71	34.96	20.96	9.37
**PRE [%]**	63.15	47.02	49.01	94.74	77.80	51.32
**TP [%]**	21.01	42.79	15.28	17.30	9.15	2.36
**FN [%]**	23.55	1.77	29.28	27.25	34.41	42.20
**FP [%]**	11.21	49.14	29.67	0.64	2.33	1.06
**TN [%]**	44.24	6.23	25.77	54.80	53.11	54.38

**Table 21:** The values of the SAST tool indicators for the Damn Small Vulnerable Web application.

	Aura	Graudit	Horusec	Bandit	ShiftLeft Scan	Semgrep
**TP**	7	0	12	11	1	9
**FN**	11	18	6	7	17	9
**FP**	0	0	2	2	0	1
**TN**	3	3	1	1	3	2
**P**	18					
**N**	3					
**TOTAL**	21					
**ACC [%]**	47.62	14.29	61.90	57.14	19.05	52.38
**SEN [%]**	38.89	0.00	66.67	61.11	5.56	50.00
**PRE [%]**	100.00	0.00	85.71	84.62	100.00	90.00
**TP [%]**	33.33	0.00	57.14	52.38	4.76	42.86
**FN [%]**	52.38	85.71	28.57	33.33	80.95	42.86
**FP [%]**	0.00	0.00	9.52	9.52	0.00	4.76
**TN [%]**	14.29	14.29	4.76	4.76	14.29	9.52

**Table 22:** The values of the SAST tool indicators for the Damn Vulnerable GraphQL application application.

	Aura	Graudit	Horusec	Bandit	ShiftLeft Scan	Semgrep
**TP**	3	0	3	3	5	3
**FN**	6	9	6	6	4	6
**FP**	1	0	2	2	0	0
**TN**	1	2	0	0	2	2
**P**	9					
**N**	2					
**TOTAL**	11					
**ACC [%]**	36.36	18.18	27.27	27.27	63.64	45.45
**SEN [%]**	33.33	0.00	33.33	33.33	55.56	33.33
**PRE [%]**	75.00	0.00	60.00	60.00	100.00	100.00
**TP [%]**	27.27	0.00	27.27	27.27	45.45	27.27
**FN [%]**	54.55	81.82	54.55	54.55	36.36	54.55
**FP [%]**	9.09	0.00	18.18	18.18	0.00	0.00
**TN [%]**	9.09	18.18	0.00	0.00	18.18	18.18

**Table 23:** The values of SAST tool indicators for the Damn Vulnerable Python Web application.

	Aura	Graudit	Horusec	Bandit	ShiftLeft Scan	Semgrep
**TP**	1	0	2	1	1	2
**FN**	2	3	1	2	2	1
**FP**	0	0	0	0	4	0
**TN**	4	4	4	4	0	4
**P**	3					
**N**	4					
**TOTAL**	7					
**ACC [%]**	71.43	57.14	85.71	71.43	14.29	85.71
**SEN [%]**	33.33	0.00	66.67	33.33	33.33	66.67
**PRE [%]**	100.00	0.00	100.00	100.00	20.00	100.00
**TP [%]**	14.29	0.00	100.00	100.00	20.00	100.00
**FN [%]**	28.57	42.86	14.29	28.57	28.57	14.29
**FP [%]**	0.00	0.00	0.00	0.00	57.14	0.00
**TN [%]**	57.14	57.14	57.14	57.14	0.00	57.14

**Table 24:** The values of the SAST tool indicators for the Tiredful API application application.

	Aura	Graudit	Horusec	Bandit	ShiftLeft Scan	Semgrep
**TP**	2	0	2	2	6	3
**FN**	6	8	6	6	2	5
**FP**	4	0	0	0	4	0
**TN**	4	8	8	8	4	8
**P**	8					
**N**	8					
**TOTAL**	16					
**ACC [%]**	37.50	50.00	62.50	62.50	62.50	68.75
**SEN [%]**	25.00	0.00	25.00	25.00	75.00	37.50
**PRE [%]**	33.33	0.00	100.00	100.00	60.00	100.00
**TP [%]**	12.50	0.00	12.50	12.50	37.50	18.75
**FN [%]**	37.50	50.00	37.50	37.50	12.50	31.25
**FP [%]**	25.00	0.00	0.00	0.00	25.00	0.00
**TN [%]**	25.00	50.00	50.00	50.00	25.00	50.00

**Table 25:** Average values of selected SAST tool indicators for applications developed using Python technology.

	Aura	Graudit	Horusec	Bandit	ShiftLeft Scan	Semgrep
**ACC [%]**	48.23	34.90	59.59	54.59	39.87	63.07
**SEN [%]**	32.64	0.00	47.92	38.19	42.36	46.88
**PRE [%]**	77.08	0.00	86.43	86.15	70.00	97.50
**TP [%]**	21.85	0.00	31.37	26.61	25.50	29.36
**FN [%]**	43.25	65.10	33.73	38.49	39.60	35.73
**FP [%]**	8.52	0.00	6.93	9.93	20.54	1.19
**TN [%]**	26.38	34.90	27.98	27.98	14.37	33.71

**Table 26:** Comparison of scan duration times. All results presented in the table are in seconds (s).

Application	Aurora	Bandit	Bearer CLI	FindSecBugs	Graudit	Horusec	Insider CLI	Phpcs-Security-Audit	Progpilot	ShiftLeft Scan	Semgrep
EasyBuggy	-	-	-	93 s	1 s	60 s	44 s	-	-	144 s	1 s
Java Vulnerable Lab	-	-	-	1 s	1 s	11 s	7 s	-	-	85 s	1 s
Security Shepherd	-	-	-	7 s	2 s	56 s	66 s	-	-	160 s	46 s
Vulnerable App	-	-	-	3 s	1 s	82 s	66 s	-	-	155 s	1 s
Broken Crystals	-	-	510 s	-	2 s	17 s	29 s	-	-	-	4 s
Damn Vulnerable Web Services	-	-	40 s	-	1 s	9 s	2 s	-	-	-	12 s
Juice Shop	-	-	148 s	-	1 s	28 s	31 s	-	-	-	92 s
NodeGoat	-	-	26 s	-	1 s	10 s	3 s	-	-	-	1 s
Conviso Vulnerable Web Application	-	-	-	-	1 s	10 s	-	1 s	1 s	89 s	1 s
Damn Vulnerable Web Application	-	-	-	-	1 s	12 s	-	1 s	3 s	72 s	1 s
WackoPicko	-	-	-	-	1 s	9 s	-	1 s	1 s	5 s	1 s
Xtreme Vulnerable Web Application	-	-	-	-	1 s	11 s	-	1 s	1 s	9 s	33 s
Damn Small Vulnerable Web	1 s	1 s	-	-	1 s	16 s	-	-	-	1 s	1 s
Damn Vulnerable GraphQL Application	12 s	1 s	-	-	1 s	14 s	-	-	-	85 s	52 s
Damn Vulnerable Python Web Application	5 s	1 s	-	-	1 s	12 s	-	-	-	108 s	63 s
Tiredful API	3 s	1 s	-	-	1 s	11 s	-	-	-	98 s	6 s
**Average**	5 s	1 s	181 s	26 s	1 s	23 s	31 s	1 s	2 s	84 s	20 s

## Data Availability

Not applicable.

## Abbreviations

The following abbreviations are used in this manuscript:

ACC	Accuracy
API	Application Programming Interface
AST	Abstract Syntax Tree
CD	Continuous Delivery
CI	Continuous Integration
CVE	Common Vulnerabilities and Exposures
CVSS	Common Vulnerability Scoring System
CWE	CommonWeakness Enumeration
DAST	Dynamic Application Security Testing
ETL	Extraction, Transformation, Loading process
FN	False Negative
FP	False Positive
IAST	Interactive Application Security Testing
MSSDL	Microsoft’s Security Development Lifecycle
N	Negative
NIST	National Institute of Standards and Technology
P	Positive
PRE	Precision
RASP	Runtime Application Self-Protection
SAST	Static Application Security Testing
SDLC	Software Development Life Cycle
SEN	Sensitivity
SSDLC	Secure Software Development Life Cycle
TN	True Negative
TP	True Positive
